# Applying deep learning to single-trial EEG data provides evidence for complementary theories on action control

**DOI:** 10.1038/s42003-020-0846-z

**Published:** 2020-03-09

**Authors:** Amirali Vahid, Moritz Mückschel, Sebastian Stober, Ann-Kathrin Stock, Christian Beste

**Affiliations:** 1 Cognitive Neurophysiology, Department of Child and Adolescent Psychiatry, Faculty of Medicine, TU Dresden, Germany; 2 Artificial Intelligence Lab, Institute for Intelligent Cooperating Systems, Faculty of Computer Science, Otto von Guericke University Magdeburg, Magdeburg, Germany

**Keywords:** Cognitive control, Decision

## Abstract

Efficient action control is indispensable for goal-directed behaviour. Different theories have stressed the importance of either attention or response selection sub-processes for action control. Yet, it is unclear to what extent these processes can be identified in the dynamics of neurophysiological (EEG) processes at the single-trial level and be used to predict the presence of conflicts in a given moment. Applying deep learning, which was blind to cognitive theory, on single-trial EEG data allowed to predict the presence of conflict in ~95% of subjects ~33% above chance level. Neurophysiological features related to attentional and motor response selection processes in the occipital cortex and the superior frontal gyrus contributed most to prediction accuracy. Importantly, deep learning was able to identify predictive neurophysiological processes in single-trial neural dynamics. Hence, mathematical (artificial intelligence) approaches may be used to foster the validation and development of links between cognitive theory and neurophysiology of human behavior.

## Abstract (web summary)

Vahid et al. use a deep-learning approach to analyze single-trial EEG data to examine theories on action control. Their approach enables the identification of spatial and temporal neurophysiological features that are predictive of the response control during the Simon task. The results confirm cognitive theory-driven approaches on the relationship between neurophysiology and human behavior.

## Introduction

The ability to monitor conflicts is an essential aspect of goal-directed behavior and action control as it allows us to select appropriate reactions in a highly complex and ever-changing world. Without this cognitive faculty, we would find ourselves to be strongly driven by sensory inputs from the external world, unable to resist distraction or deal with ambiguous/contradictory information. Given this high relevance, several major theoretical frameworks have been setup to explain these and related processes of action control^1–4^.

Experimentally, (response selection) conflicts that require action control are often examined using paradigms like the Flanker or Simon task^5^. In these paradigms, people carry out a choice task on stimuli that have a task-relevant stimulus feature determining the required response and (at least one) irrelevant stimulus (feature). The latter can facilitate the selection of the correct response by activating the same response as the relevant stimulus feature (non-conflict trial), but it can also diminish the ability to select the correct response by eliciting a response tendency other than the correct response (conflict trial)^5^. The general finding in this context is a “conflict effect” that is indicated by slower and/or more error-prone responses in conflicting trials, as compared to non-conflicting trials^6^. In the Simon task, stimulus-response conflicts requiring action control occur due to the incongruent lateralization of the stimulus and the responding hand^5,7–9^. Considering the cognitive processes involved, there are (at least) two major streams of research on how this conflict comes about^10^: One stream refer to the role of attention and spatial coding processes, the other focuses on mechanisms related to intentional response selection processes. The reason is that the type of conflict being measured in Simon tasks is a so-called stimulus-response (S–R) conflict^5,10^. Thus, both stimulus-related (attentional) and response-related processes seem to play a role.

In the last two decades, a lot of neuroscientific research using fMRI, EEG, and computational methods has been carried out in order to identify and elucidate the neural correlates of conflict monitoring processes during action control^11–14^. Considering EEG data, S–R conflicts are associated with modulations in the time window of the N2 ERP component at frontal and fronto-central electrode sites^12,15–26^. Similarly, processes of motor activation, like lateralized readiness potentials, are modulated^27,28^. Also, attentional selection processes and neurophysiological correlates of attention (like the N1)^29^ and spatial attention (N2pc) have been shown to be modulated in the Simon task^27,30^. This seems reasonable given the importance of attentional (orienting) processes in the Simon task, which requires the integration of distinct and spatial stimulus position (codes)^10^.

Yet, only very few studies have also reported linear relationships between the amplitude of the above-mentioned neurophysiological correlates and task performance^13^. The inter-relation between behavior and associated neurophysiological dynamics is hardly strictly linear, albeit most analysis approaches in cognitive neuroscience rely on the assumption of linearity when applying (correlational) approaches to connect behavioral data and neurophysiological data. Generally, there is rarely a one-to-one relationship between EEG-derived neural signals and behavior^31^, although this is often suggested, or at least implied. Furthermore, the neurophysiologic data used for the formation of ERPs is inherently noisy. Therefore, it is substantially harder to establish such functional connections at the single-subject level or the single-trial level^31–33^. This problem severely limits the degree and level at which neural signatures may be functionally related to human behavior^31^, or indicate cognitive processes involved in a specific situation (e.g., conflict processing). These shortcomings may be tackled with machine learning methods^31^. There are already first encouraging approaches, as more conventional machine learning approaches like support vector machines (SVMs) have been successfully applied in comparable contexts^34–38^. Still, one major shortcoming of these conventional SVM approaches is that even though the included “features” (i.e., EEG signatures) may be selected by algorithms^39^, SVMs can only handle a small number of features at a given time/analysis^40^. As only some aspects or timepoints of the EEG data can therefore be considered in feature extraction via SVM, these approaches cannot appropriately account for the time information/dimension of the neurophysiologic data. Yet, this is particularly critical with EEG data, where timing properties of neurophysiological processes are important to consider. This means that possibly predictive/behaviorally relevant aspects of neural processes may still remain unnoticed in SVM approaches. In contrast to this, deep learning allows computational models to learn representations of data with multiple levels of abstraction^41^, thus truly using all of the information that the dataset has to offer^40,42^. This is a major advantage over more conventional machine learning approaches. So far, only a small number of studies have used deep learning for the classification of EEG data^43–46^. Likewise, to our knowledge, there is no study applying deep learning methods in a cognitive control context to examine the usefulness of single-trial EEG data for the prediction of the trial type (i.e., conflicting and non-conflicting trials in an experiment) and associated differences in cognitive processes. It is, to our knowledge, the first EEG study using deep learning to characterize the processes underlying action control in conflict tasks on a single-trial level and shows how this data-driven deep-learning approach can be used for hypothesis generation, confirmation of current theory, and for practical applications demanding high accuracy.

Importantly, we do so in a theoretically meaningful manner by integrating a “saliency map” approach^46,47^. This is necessary from a cognitive perspective, because it is crucial to know which aspects of the neurophysiological data contribute most to classification performance. To learn which EEG input features (timepoints/channels) have the highest impact on the classification decision, we employed a “saliency map” approach^46,47^. Using this approach, it is possible to delineate which timepoints and electrode sites in the EEG contribute most to classification accuracy; i.e., the correct identification/classification of trial type (the combination of trial conflicts and the responding hand) on the basis of EEG data. This is an important aspect considering the ultimate goal of informing cognitive neuroscience theory by using deep learning methods. Given that cognitive sub-processes are reflected in specific EEG data time windows, it is reasonable to hypothesize that a purely data-driven approach such as deep learning should identify (but not necessarily be limited to) EEG features that correspond to ERP correlates. This will have major consequences: If a purely mathematical procedure (i.e., deep learning), which runs without any strong assumptions (e.g., without being informed about relevant EEG features reported in literature and cognitive theories), identifies aspects in neurophysiological signals that are considered relevant in the context of psychological theory formation, this will demonstrate that data-driven artificial intelligence methods can strongly contribute to the validation and further development of cognitive concepts. This will considerably contribute to how deep learning methods are seen in cognitive neuroscience. Moreover, if such classifications are possible using single-trial EEG data, this will represent an important step towards going beyond conventional ERP components and to functionally relate EEG features to behavioural performance. Most noteworthy, this would be done at the time scale of single trials, i.e., the neurophysiological processes that are directly associated with a given single response^31^.

## Results

The EEG was recorded during a Simon Task (see methods section for details), which was previously used to examine conflict monitoring processes during action control^28,48–50^. In each trial, the target stimulus (capital letter A or B) was presented for 200 ms in one of two white frame boxes place on the left or right of centrally presented fixation cross. In the other white frame box, a noise stimulus (three horizontal white bars) were presented simultaneously. The left key had to be pressed when the letter “A” was presented, the right key had to be pressed whenever the letter “B” was presented. Trials in which the target stimulus and the location of the stimulus matched (i.e., when A was presented in the left and B in right white frame box) were non-conflicting trials. The other target identity and location combination represented conflicting trials. Thus, there were four classes of trials: (i) left hand non-conflict trials, (ii) left hand conflict trials, (iii) right hand non-conflict trials, and (iv) right hand conflict trials.

### Behavioral data

We conducted separate repeated measures ANOVAs of the error rates (i.e., incorrect button press) and correct RT data using the within-subject factors “hand” (left vs. right) and “conflict” (conflicting vs. non-conflicting). Descriptive data are provided as mean ± SEM. For error rates, we found a main effect of hands (F(1,185) = 5.07; *p* = .025, $\eta _p^2$ = .027; left hand = 6.18 % ± 0.3; right hand = 6.79 % ± 0.4), the Simon effect, as indicated by a main effect of conflict (F(1,185) = 218.61; *p* < .001, $\eta _p^2$ = .542; conflict = 9.6 % ± 0.5; non-conflict = 3.4 % ± 0.3), and an interaction of hand × conflict (F(1,185) = 41.79; *p* < .001, $\eta _p^2$ = .184). Post hoc *t*-tests revealed that there were significant Simon/congruency effects (i.e., less errors in congruent than in incongruent trials) for both hands (all *t* ≥ 10.80; all *p* < .001). Yet, the Simon effect (i.e., the difference between non-conflicting and conflicting trials) was significantly larger (i.e., more negative) for right hand responses (7.6 % ± 0.5) than for left hand responses (4.7% ± 0.5) (*t*(185) = 6.46; *p* < .001). Lastly, there was a dissociation effect of responding hand, which differed between the two task conditions: In congruent trials, participants showed significantly less errors with the right hand (3.0 % ± 0.3), than with the left hand (3.8 % ± 0.3) (*t*(185) = 3.80; *p* < .001). In incongruent trials, we found the opposite, namely significantly more errors with the right hand (10.6 % ± 0.6), than with the left hand (8.6% ± 0.5) (*t*(185) = −4.59; *p* < .001).

For correct RTs, we ran a comparable ANOVA and found a main effect of hands (F(1,185) = 15.83; *p* < .001, $\eta _p^2$ = .079; left hand = 414 ms ± 3; right hand = 409 ms ± 3), the Simon effect, as indicated by a main effect of conflict (F(1,185) = 776.45; *p* < .001, $\eta _p^2$ = .808; conflict = 430 ms ± 3; non-conflict = 393 ms ± 3), and an interaction of hand x conflict (F(1,185) = 11.86; *p* = .001, $\eta _p^2$ = .060). Post hoc *t*-tests revealed that there were significant Simon/congruency effects (i.e., better performance in congruent than in incongruent trials) for both hands (all *t* ≥ 20.657; all *p* < .001). Yet, the Simon effect (i.e., the difference between non-conflicting and conflicting trials) was significantly larger for right hand responses (41 ms ± 2) than for left hand responses (34 ms ± 2) (*t*(185) = 3.44; *p* = .001). Lastly, there was a dissociation effect of responding hand, which differed between the two task conditions: In congruent trials, participants showed significantly better performance with the right hand (388 ms ± 3), than with the left hand (397 ms ± 3) (*t*(185) = 5.18; *p* < .001). In incongruent trials, we found no such difference (*t*(185) = 0.92; *p* = .359).

### Deep learning predicts the presence of conflicts using EEG data

**Fig. 1:**
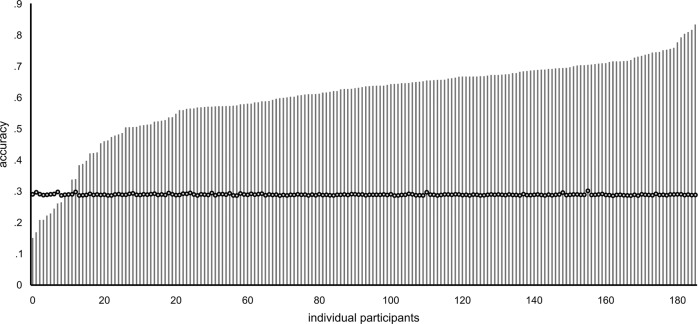
Classification accuracy for this 4-class problem is shown for each individual subject. On the *x*-axis the individual subjects are shown. The *y*-axis denotes the classification accuracy for the 4-class problem in each individual. The dotted lines denotes the chance level calculated for the individual participant using the method by Combrisson and Jerbi^53^.

Since the behavioral data revealed that the responding hand also modulated behavioral performance, this factor was also considered in the deep learning step. Therefore, the main study question was based on a 4-class problem: We used single-trial EEG data from (i) left hand non-conflict trials, (ii) left hand conflict trials, (iii) right hand non-conflict trials, and (iv) right hand conflict trials to train the deep learning architecture (EEGNet^51^) on a training dataset. The trained model was then applied to the test/validation dataset in order to see how well it could correctly identify the four different conditions. That is, for evaluating the classification performance, we use the “leave one out subject” (LOOS)-approach^52^ (see methods section for details). We examined both of (4,2) and (8,2) options for the number of temporal and spatial filters in EEGNet and the averaged classification accuracy among subjects are 56% and 60%, respectively. Since the accuracy for (8,2) is higher than (4,2), in the rest of the result section, we only focus on the model based on (8,2). In Fig. 1, the classification accuracy for this 4-class problem is shown for each individual subject.

**Fig. 2:**
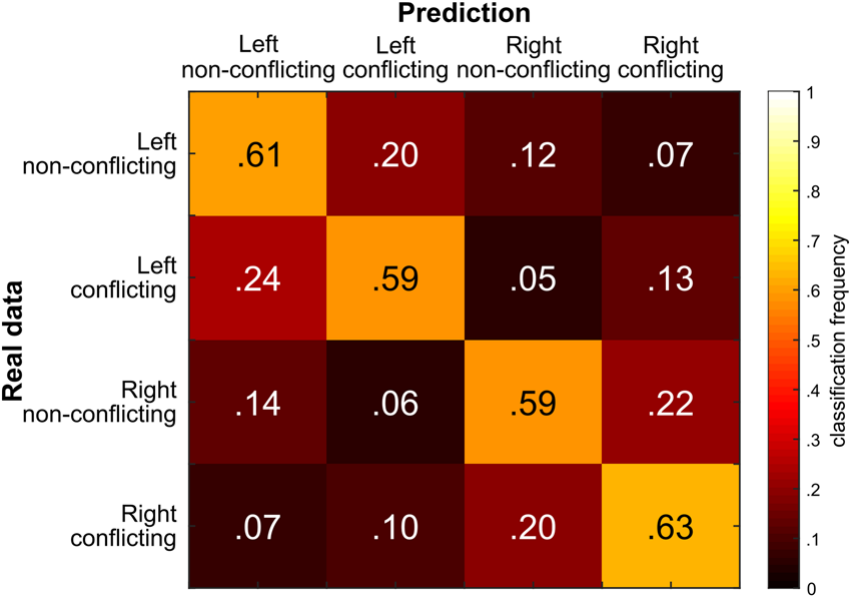
Confusion matrix showing the classification results for the different conditions. Colour shadings and numbers in the matrix denote the frequency at which the real data (“true”) label was classified into one of the four possible predicted classes.

In an infinitely sized dataset, the chance level of such a 4-class problem would be at 25% classification accuracy. As the number of samples was of course finite in our dataset, the number of correct trials varied slightly between conditions and subjects. As a consequence, the chance level also slightly varies between subjects^53^. The subject-wise chance level was determined using the method by Combrisson and Jerbi^53^ and is also shown in Fig. 1. The mean single-subject chance level was 28.88% (SD = 0.02). As explained in the methods section, we calculated a threshold that indicates classification accuracies well above chance level by assuming that classification error obeys a binomial cumulative distribution^46^. Combrisson and Jerbi^53^ have shown that this method shows no difference to permutation testings when sample sizes are *N* > 100. Since this was the case in the current study, we refrained from permutation testing^46^, which would have required re-estimating the EEGNet model 1000 times. Figure 1 shows that the EEGNet prediction of the trial class was above chance level in *N* = 175 subjects (i.e., 95.59% of subjects). The average accuracy of trial class prediction on the basis of the single-trial EEG data in these *N* = 175 subjects was 60.1% (SD = 12.9), and thus 33.36% (SD = 9.22) higher than the individual chance levels (*t*(174) = 48.24; *p* = 1.21e^−102^). It was further shown that the number of trials available for deep learning increased the classification accuracy (r = .165; *p* = .014), but this effect was small and only explained 2% of the variance in classification accuracy (R^2^ = .02). The confusion matrix for the 4-class problem is shown in Fig. 2. Rows show the real (“true”) label, the columns show the label, which was predicted on the basis of the single-trial EEG data.

**Fig. 3:**
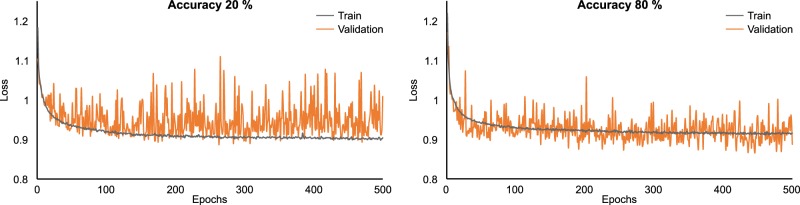
Training and validation loss for two subjects. One with high accuracy (80%) and one with low accuracy (20%).

As can be seen in the confusion matrix (Fig. 2), the average prediction accuracy of the EEGNet was ~60% (see diagonal from top left to bottom right in the confusion matrix). It was hence not only above chance level, but also substantially larger than the percentage of incorrect predictions. For example, right hand conflict trials were only incorrectly classified as left hand conflict trials in 7% of cases. Opposed to this, right hand conflict trials were correctly classified as such in 63% of the cases. Generally, the confusion matrix shows that the taken deep learning approach is well able to classify trial class (experimental condition) on the basis of the single-trial EEG data. Figure 3 shows the training and validation loss versus epochs (i.e., duration of training) for two subjects—one with high accuracy (i.e., 80%) and one with low accuracy (20%). In all of the test subjects, the lowest cross-entropy loss in the validation set happens before the last epoch (500) and after ~100 epoch. As shown in Figure 3, the validation loss is very noisy and it has a decreasing trend roughly until epoch 20, but after that, it oscillates. To avoid overfitting we saved the model at the epoch with lowest cross-entropy loss in the validation set, as suggested in the original work introducing EEGNet.

To examine whether the chosen deep learning approach does indeed reveal the ‘best’ solution to the problem and to check whether more simple machine learning approaches (e.g., support vector machines, SVMs) are able to perform at a similar level, we re-run the entire classification procedure using an SVM approach (please refer to the methods section for details). The classification accuracy for the SVM approach was 32%, which is very lower than classification based on EEGNet approach.

### Attention and response selection processes are predictive

**Fig. 4:**
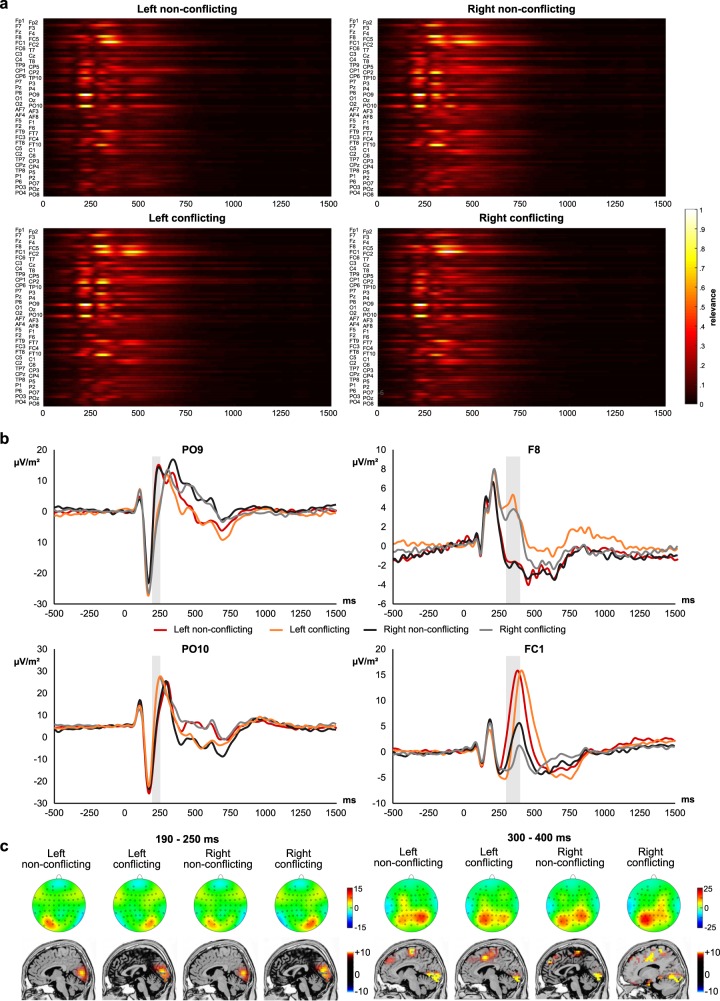
Visualization of the deep learning analysis. **a** Visualization maps showing the relevance of all timepoints and electrodes for classification between the four different classes of trials. Values close to 1 indicate that the specific feature at the specific timepoints contributes most to classification accuracy. The *x*-axis denotes the time in ms after target stimulus presentation. The *y*-axis denotes the different electrode sites. **b** Event-related potential at the electrode sites contributing most to classification accuracy in the deep learning model. The *x*-axis denotes the time in ms after target stimulus presentation. The *y*-axis denotes the voltage (note that the scaling of the *y*-axis differs between the plots). The gray-shadings denote the time interval that was found to contribute strongly to classification performance in the deep learning network. **c** The scalp topography plots (top) are shown denoting the distribution of amplitudes across the scalp in the time interval that contributed most to classification performance. For the sensor space images the ‘top-view’ is presented. Hence, electrodes appearing on the left in the figure are also place at the left of the scalp. Red colours denote positive amplitudes, blue colours negative amplitudes. At the bottom, the corresponding source localization results are shown for each of the different conditions. Only significant activations are shown (*p* < .05) corrected for multiple comparisons using voxel-wise randomization tests with 2000 permutations and statistical nonparametric mapping procedures (SnPM).

Figure 4 presents separate visualization (“saliency”) maps for each of the four classes. As can be seen in Fig. 4, parietal-occipital electrodes (PO9 and PO10) strongly contributed to classification accuracy in the time window from 190 ms to 250 ms. Importantly, this was the case for all four classes of trials. The event-related potential (ERP) plots showing activity at electrodes PO9 and PO10 are given in Fig. 4. As can be seen in Fig. 4, the identified time window overlaps with the N1 ERP component, which is known to reflect attentional selection processes^29,33^. The sLORETA analysis in this time window shows that in all four experimental conditions, areas in the occipital cortex, especially the cuneus (BA17 and BA18), were activated.

However, Fig. 4 also shows that activity at electrodes F8 and FC1 was highly relevant for classification accuracy, especially in the time window between 300 and 400 ms after target stimulus presentation. This combination of topography and timing may be attributed two ERPs that are traditionally associated with performance and conflict monitoring in the Simon task: One of them is the N2, which has frequently been reported to be relevant during S–R conflicts measured in the Simon task^12,15,17–20,22–24,26,54^. The other is the lateralized readiness potential (LRP), which reflects lateralized, motor response-related activations of the lateral vs. contralateral motor cortex, SMA, and adjacent brain areas in unilateral responses^27^. The sLORETA analysis in this time window shows that in all four experimental conditions, areas in the superior frontal gyrus (BA6) and medial frontal gyrus (BA24) were activated aside visual cortical regions (BA17 and BA18).

## Discussion

In this study, we tested whether deep learning, which is a purely mathematical procedure, can identify neurophysiological correlates of cognitive processes that are commonly considered relevant in the context of psychological theory formation on action control, especially in the context of conflicts. Moreover, if such classifications are possible using single-trial EEG data, this will represent an important step towards going beyond conventional ERP components and to functionally relate EEG features to behavioral performance. Most noteworthy, this would be done at the time scale of single trials, i.e., the neurophysiological processes that are directly associated with a given single response^31^.

The results show that deep learning allows to classify different classes of trials in an action control task on a single-subject and, even more importantly, on a single-trial level. This was possible in more than 95% of the included participants and classification accuracy was ~33% above chance level. In the remaining 5% of subjects, no classification of trials above chance level was possible. We used a saliency map approach to determine which EEG features contributed most to this high classification accuracy. This showed that activity at posterior electrodes in the N1 ERP time window strongly contributed to classification accuracy. Source localization further showed that this was associated with activation differences in the cuneus (BA17, BA18). Of note, these areas have been associated with attentional selection processes reflected by the N1^29,55^. N1-related processes have been shown to be of special functional importance in the Simon task^30^. It has been proposed that these attentional processes are relevant because the performance in the Simon task requires different stimuli signaling for distinct responses to be integrated with the spatial position (location) of these stimuli^10^. To form such spatial codes, it has been suggested that attention needs to be moved to the target’s location^56^. Indeed, it has been shown that when there is no shift of attention, there is also no Simon effect^56^. The finding that the applied deep learning method detects these processes, shows that attentional processes are key to the understanding of condition-induced differences in cognitive sub-processes occurring during the Simon task. However, it should be noted that the current experimental setup is not able to dissociate between spatial coding approaches and attention-shifting approaches, because the design of the experiment included bilateral presentation of visual stimuli. The key difference between spatial coding approaches and attention-shifting approaches is that only the latter assumes that it is not the location of the stimulus that matters for coding but the direction into which attention is shifted before processing that stimulus. Only with unilateral presentation of visual stimuli, one can test these two approaches against each other. With respect to theoretical concepts explaining cognitive mechanisms underlying the Simon task, the deep learning results show that attention and the attention-shifting approach to spatial stimulus coding^10,56^ have a strong explanatory power for the Simon effect. Most noteworthy, this is a case where a purely mathematical procedure identified exactly those aspects of the neurophysiological signal that are already considered relevant in the context of psychological theory formation.

Additionally, it was shown (cf. Fig. 3) that activity at electrode F8 and FC1 was also highly relevant for classification accuracy, especially in the time window between 300 and 400 ms after target stimulus presentation. Of note, this combination of topography and timing may be attributed two ERPs that are traditionally associated with performance and conflict monitoring in the Simon task. One of them is the N2, which has frequently been reported to be relevant during S–R conflicts measured in the Simon task^12,15,17–20,23,24,26,54^, and likely reflects conflict monitoring and the associated cognitive (need for) effort^13^. The other component at this time and topography is the movement-related potential, which reflects different activations of the lateral vs. contralateral motor cortex, SMA, and adjacent brain areas in unilateral responses^27^. In short, the activation difference between the two hemispheres, which can also clearly be seen for electrode FC1 (see Fig. 3), is thought to reflect lateralized motor response activation. In this context, medial frontal structures, superior frontal structures, and supplemental motor areas have been shown to play an important role in conflict processing^9,28,57–60^. Albeit EEG source estimations are not as precise as functional imaging to localize neural activity, which is a limitation of the applied methods, the sLORETA analysis for this time window showed that areas in the superior frontal gyrus (BA6) and medial frontal gyrus were activated in all four experimental conditions. This corroborates the above interpretations that response selection and control processes (i.e., response codes) play an important role for Simon task performance^10^. As previously mentioned, the conflict evoked in the Simon task is a stimulus-response (S–R) conflict, which arises from the mismatch between stimulus location and motor effector (responding hand) location. Additional control demands are required for correct sensorimotor transformation in conflicting trials, as the interference caused by the incorrect response activation (partly in the “wrong” hemisphere) needs to be resolved^61,62^.

Taken together, the deep learning results show that neurophysiological correlates of both attentional processes in occipital areas and response selection processes in frontal areas exhibit distinct markers that strongly contribute to the correct classification of trial type in the Simon Task, which we used as a means to examine action control/conflicts. The data hence provide evidence for theories stressing the functional relevance of perceptual/attentional processes, as well as for theories stressing the functional relevance of response selection/conflict monitoring processes in the Simon task. Intriguingly, influential theoretical frameworks like the ‘Theory of event coding (TEC)’^2^ propose that both perception and action are processed at the same representational level and by using the same kinds of codes. To-be-produced events (i.e., actions/responses) and perceived external events (i.e., stimuli) are coded for by their constituting feature codes within a common format—the ‘event file’^63^. Stimuli (e.g., letters) are coded by objective features, such as their shape, colour and identity (i.e., A, B etc.). These features are closely bound to one another (i.e., integrated) to achieve a coherent perception. Likewise, responses are represented by features detailing a potential outcome, e.g., the required hand movement. As for the Simon effect, it has been proposed that task-relevant stimulus features (i.e., letter identity) and task-irrelevant features (i.e., stimulus location) are bound together in one representation/code^10^ and the generated responses are additionally bound within the same representation/code. Thereby, the TEC highlights that both stimulus and response processing are essential for the Simon task effect and conflict processing during action control. Importantly, both of these aspects were also identified as most relevant by the deep learning approach. The crucial point is that a deep learning procedure is able to identify almost exactly those aspects of the neurophysiological signal that are considered most relevant for psychological theory formation. Moreover, this represents an important step towards going beyond conventional ERP components, as was be done at the time scale of single trials, i.e., the neurophysiological processes that are directly associated with a given single response^31^. This suggests that cognitive-theoretical concepts can be validated by applying deep learning procedures to neurophysiological data. Likewise, deep learning might prove fruitful in contexts where there are no predetermined concepts or hypotheses. In this respect, data-driven methods may strongly contribute to the validation, but also support the further development of theoretical concepts in cognitive control. Similarly, deep learning may foster the development of links between cognitive theory and neurophysiology in the future.

## Materials and methods

### Sample, sampling strategy, and data collection

*N* = 186 healthy adult volunteers between 18 and 34 years of age (mean 23.7, SD = 3.0) participated in the study. *N* = 106 of them were females. Participants were recruited from the TU Dresden using voluntary panel board announcements and received course credits or a financial compensation for participation (€ 15). All participants had normal or corrected-to-normal vision. No participant reported a history of neurological and psychiatric illness. The study was approved by the Ethics Commission of the Medical Faculty of the TU Dresden and the Ruhr-Universität Bochum and all participants provided written informed consent. No participants dropped out or declined for personal reasons. In the study, an EEG-deep learning approach was used on which depends on the available EEG data points in the entire sample. Single-trial EEG data were used. Thus, the number of data points are: number of subjects × electrode number × sampling rate × length of the EEG intervals analysed × number of EEG epochs analyzed. For the current study, this means that ~3,348,000,000 data points were available for the deep learning procedure using the EEGNet architecture. No data were excluded from the analysis. No participants dropped out or declined for personal reasons. It was a complete within subject design and there was no allocation of subjects to experimental groups. The data were collected between July 2011 and November 2012.

### Task

**Fig. 5:**
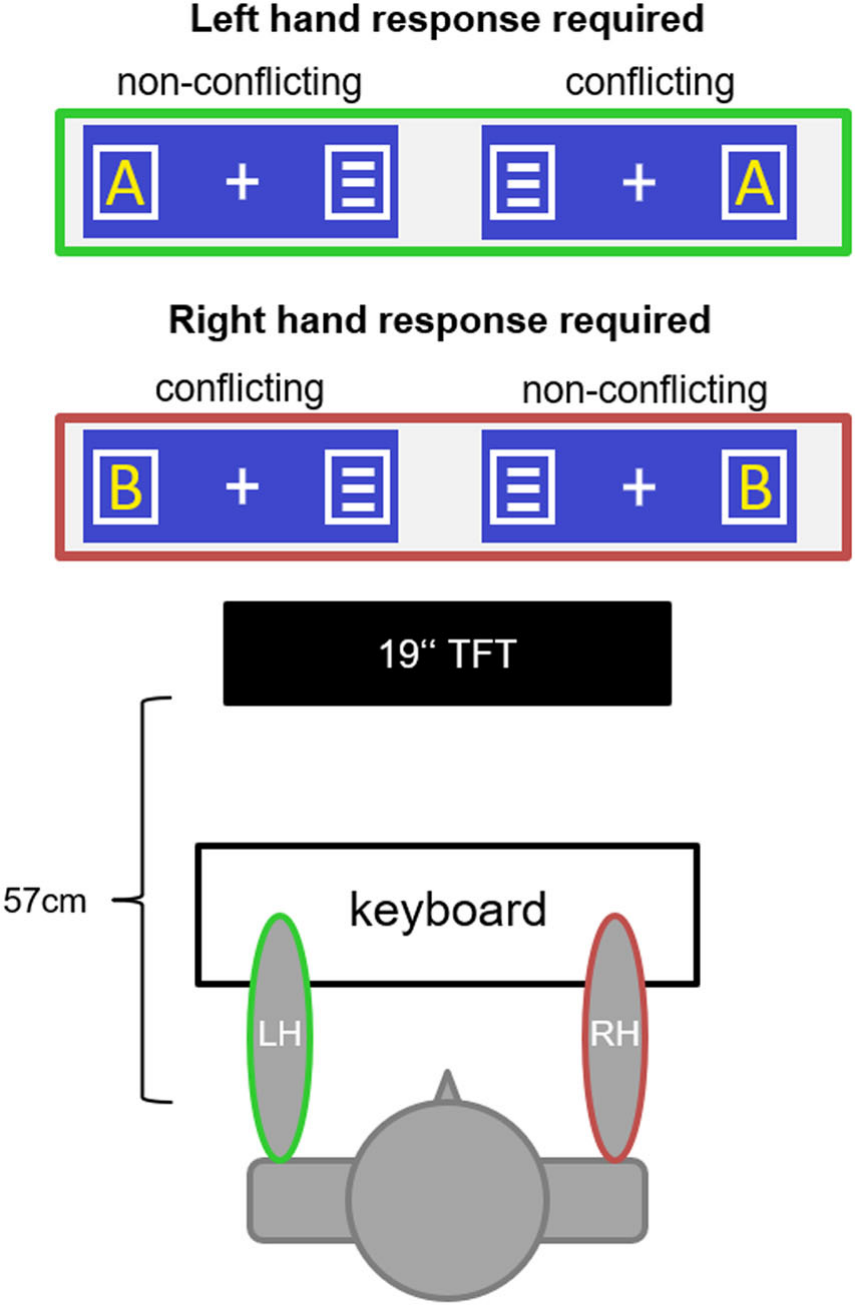
The target stimuli (letters) could be located in either of the boxes on the left or the right of the fixation cross. Letter A required a reaction of the left hand (irrespective of the spatial position of the letter) while letter B required a reaction of the right hand (irrespective of the spatial position of the letter).

The software “Presentation” (version 14.9. by Neurobehavioral Systems, Inc.) was used for stimulus presentation, response recording, and sending the EEG triggers. We used a standard Simon task (Fig. 5), which was previously used in other, unrelated studies to examine conflict monitoring processes during action control^28,48–50^.

Participants were seated at a distance of 57 cm in front of a 19” TFT screen presenting a white fixation cross and two white frame boxes on a black background. The fixation cross was in the center of the screen and the white frame boxes were located 1.1 degrees visual angle to the left and right of the fixation cross. In each trial, the target stimulus (capital letter A or B) was presented for 200 ms in of the white frame boxes. In the other white frame box, a noise stimulus (three horizontal white bars) were presented simultaneously. Responses were carried out on the QWERTZ keyboard and participants were asked to press the left or right CTRL key. The left key had to be pressed when the letter “A” was presented, the right key had to be pressed whenever the letter “B” was presented. The responses were carried out using the index finger. Each trial was terminated by the first button press after target onset. To ensure speeded responding, a speed-up sign was presented whenever participants failed to respond within 500 ms after target onset. If no response was given in a trial, the trial was terminated 1700 ms after target stimulus presentation and coded as a “miss”. Response-stimulus intervals (RSI) randomly varied between 2000 ms and 2500 ms. The experimented consisted of 400 trials equally divided in conflicting and non-conflicting trials in which the response was given using the left or the right hand. Trials in which the target stimulus and the location of the stimulus matched (i.e., when A was presented in the left and B in right white frame box) were non-conflicting trials. The other target identity and location combination represented conflicting trials.

### EEG recording and preprocessing

The EEG was continuously recorded from 60 Ag/AgCl electrodes mounted in an elastic cap (EasyCap Inc.) while subjects performed the task using a BrainAmp amplifier (Brain Products Inc.) (500 Hz sampling rate, filter band-width 0.3–80 Hz). During recording, the electrode impedance was below 5 kΩ. Electrode Fpz served as reference electrode. Offline, the EEG data were inspected for gross technical artifacts using the Brain Vision Analyzer 2 software package (Brain Productions Inc.). EEG periods with gross technical artifacts (i.e., offsets in the EEG) were marked (cut-out). Also, channels with no activity (‘flat line’ channels) were discarded from the EEG. Then a band-pass filter from 0.5 to 20 Hz was applied (48 dB/oct). After that, an independent component analysis (ICA, infomax algorithm) was run to identify horizontal and vertical eye movements, as well as artifacts. These artifacts were corrected in the EEG. Thereafter, previously discarded ‘flat line’ channels were interpolated. After these preprocessing steps, the data were segmented. For that only trials with correct responses were used. There were four segment classes: (i) left hand non-conflict trials, (ii) left hand conflict trials, (iii) right hand non-conflict trials, and (iv) right hand conflict trials. The segments lasted from 100 ms pre-stimulus onset to 1500 ms post-stimulus onset, resulting in a total interval length of 1600 ms. Within these single-trial segments, an automated artifact rejection procedure was performed applying the following criteria: (i) maximally allowed voltage step 50 μV/ms; (ii) maximally allowed difference of values in 200 ms intervals of 200 μV; (iii) lowest allowed activity in 100 ms intervals of 0.5 μV. The remaining segments were then subjected to a current source density transformation, which results in a reference-free representation of the data and acts as a spatial filter^64^. In a final preprocessing step, the pre-stimulus baseline was set from −100 ms to 0 ms before stimulus onset. These single-trial data from time point zero to 1500 ms after target presentation were used for deep learning.

### Deep learning

For deep learning, we used the EEGNet architecture^51^. The EEGNet architecture can be downloaded from https://github.com/vlawhern/arl-eegmodels. The procedure and the deep learning architecture used in the current study is almost identical to a previous study by our group^46^. Originally, the deep learning architecture (EEGNet) has been developed to decode brain states in Brain Computer Interfaces. Its performance has already been investigated using various event-related potential datasets^51^. The parameters for each layer of the deep learning network used in the current study are described in Table 1.

**Table 1:** Details of the EEGNet architecture used to classify single-trial EEG data.

Block	Layer type	Filters	Size	Parameters	Output dimension	Activation	Mode
1	Input				(C,T)		
	Reshape				(1,C,T)		
	Conv2D	*F*_1_	(1,64)		(*F*_1_,C,T)	Linear	Same
	BatchNorm			2∗*F*_1_	(*F*_1_,1,T)		
	DepthwiseConv2D	D∗*F*_1_	(C,1)	C∗D∗*F*_1_	($D\ast F_1$,1,T)	Linear	Valid
	BatchNorm			2∗D∗*F*_1_	($D\ast F_1$,1,T)		
	Activation				($D\ast F_1$,1,T)	ELU	
	AveragePool2D		(1,4)		($D\ast F_1$,1,T/4)		
	Dropout				($D\ast F_1$,1,T/4)		
2	SeparableConv2D	*F*_2_	(1,16)	16∗D∗$F_1 + F_2\ast (D + F_1)$	(*F*_2_,1,T/4)	Linear	Same
	BatchNorm			2∗*F*_2_	(*F*_2_,1,T/4)		
	Activation				(*F*_2_,1,T/4)	ELU	
	AveragePool2D		(1,8)		(*F*_2_,1,T/32)		
	Dropout				(*F*_2_,1,T/32)		
	Flatten				(*F*_2_∗T/32)		
	Dense	($2\ast F_2$∗T/32)			N	Softmax	

The EEGNet architecture is identical to a previous study by our group^46^.

*C* number of channels, *T* number of timepoints, *F*_*1*_ number of temporal filters, *D* number of spatial filters, *F*_*2*_ F1∗D, *N* number of classes, respectively.

To apply EEGNet, one needs to create two dimensional arrays from single-trial EEG data in which channels (C) and time (T) are represented in columns and rows, respectively. Consequently, the input has a shape (C,T). EEGNet has two main blocks (cf. Table 1): The first block produces temporal feature maps by applying convolutional filters. The convolutional filters have a width of 64 samples. Thereafter, D spatial filters spanning all EEG channels were learned by applying depths-wise convolution. This was done for each temporal feature map. Depth-wise convolution is connected just in one previous feature map and D is a parameter that controls the number of spatial filters the model must learn for each temporal filter. This is why for each temporal feature map, D spatial filters have to be employed. After applying temporal and spatial filters, batch normalization followed applying an exponential linear unit (ELU) as an activation function. This included average pooling over 4-time steps with stride of 4. This resulted in outputs with the shape of (F1∗D,1,T/4). In the second block, a separable convolution consisting of depth-wise temporal filters of width 16 followed by a point-wise convolution was used. Since separable convolution has fewer parameters than ordinary convolution, the model is less prone to overfitting. Again, batch normalization followed by ELU activation function, average pooling over 8-time steps and dropout were applied thereafter. Finally, the classification step is done using a dense layer with a softmax-activation function.

In the current study, we investigate in how far the single-trial neurophysiological data at the single-subject level enables a classification of trials into (i) left hand non-conflict trials, (ii) left hand conflict trials, (iii) right hand non-conflict trials, and (iv) right hand conflict trials. EEGNet was applied as a classifier to decode brain cognitive states. For evaluating the classification performance, we use the “leave one out subject” (LOOS)-approach^52^. Using the LOOS method, the amount of data in the test set is equal to number of trials that a subject performed. Therefore, this method is different from “leave one out” (LOO), which just considers one data in test time. Importantly, problems that have been discussed^52^, such as maximizing variance of the test set or overfitting, are less of an issue in LOOS. In this approach, one subject is selected for testing, while the remaining subjects are used for training the classifier. Four subjects also are randomly selected for validation sets among training subjects in order to use for early stopping. The process of selecting one subject as testing and others as training continues until all of subjects are selected as testing subject one time. We trained the model for 500 epochs and saved the model with lowest cross entropy in validation set. As suggested in original paper for number of temporal and spatial filters (F1,D) two option were employed, i.e., (4,2) and (8,2). Moreover, the batch size is set to 32. To train EEGNet, the ADAM optimization was used^65^. Since the number of trials varies among subjects and conditions, our datasets are unbalanced, and we apply a class weight which is the inverse of the proportion in the training data, with the majority class set to one. To evaluate the model’s performance, we report the entire confusion matrix and accuracy (see results section).

In order to investigate what kind of features (i.e., single timepoints in single channel) have a stronger impact on model’s classification decision, we used the “saliency map” approach^47^. Goal of such saliency maps is to find features in each individual single-trial data that have highest impact on classification output. For the calculation of a saliency map, one needs to take the gradient of the classification score, i.e., before applying the softmax-activation function to the input data. This map provides information how much the model’s output change when there are small changes in the input data on the single-subject level. For visualization, all saliency maps of every single-trial belonging to a class were averaged and are shown in the results section. In order to have more obvious visualization map, we also performed a normalization step in which the minimum and the maximum of averaged saliency map scores is set to 0 and 1, respectively. Using this scale, values close to 1 indicate that this feature/time point strongly contributes to classification accuracy. Importantly, and to ensure that the model’s classification performance in the 4-class problem is significantly above chance level for each single-subject, we calculated a threshold indicating classification accuracies significantly above chance level by assuming that the classification error obeys a binomial cumulative distribution^53^. We used the MATLAB function “binoinv” to compute the statistically significant threshold according to

$${\mathrm{std}}\left( \alpha \right) = {\mathrm{binoinv}}\left( {1 - \alpha ,n,\frac{1}{c}} \right) \ast \frac{{100}}{n}$$

for each single-subject. In this formula, α is the significance level, n is number of predictions (i.e., number of data in test set) and c is number of classes. This function provides a threshold which means that a classification accuracy higher than this threshold is significantly above chance level. The binomial method has some advantages over other methods such as permutation tests for investigation classification performance statistically. Permutation tests are very time consuming because the model needs to be trained several times (e.g., 1000 times) and running a deep learning architecture such as EEGNet based on LOOS for 1000 times is not practical. Importantly, it has been shown that when the number of trials to predict is more than ~100 there is not relevant difference between permutation testing and the binomial approach^53^. Since the number of samples in the test set is equal to the number of trials that a single-subject performed (*N* = 346 ± 30 in the current study), the binomial result is valid.

However, of course there are also other DNN methods suitable for EEG data^43,44^. Bashivan et al.^43^ proposed a deep learning architecture for the classification of EEG data in a working memory task. This study entirely focused on the frequency spectrum, which is calculated based on FFT. However, for the purpose of the current study, the data structure must be visualizable to be able to compare with previous finding using standard EEG methods and to be able to inform cognitive theory, which have been well connected with standard EEG methods. The approach by Bashivan is based on frequency information, which is not the purpose of the current study. For the current study, the time information is very important, which is not evident when focusing on the frequency spectrum of the EEG. Moreover, the approach proposed by Bashivan et al. results in a data structure, which is hard to visualize: After a few processing steps, each trial is divided into 7-time frames and for each of them an EEG image is constructed, making the entire dataset a video. This video like data structure can capture information in EEG data and it is well suited for applying DNN architectures that are designed for video or image. However, since there are some transformations on raw EEG data (i.e., time and channel), the visualization of the model is not straight forward. Interpolation is employed on 2D channels. Because of the interpolation over channels in this method, we do not know which channels exactly are more important for classification. This is, however, is important to connect to existing research using standard ERP methods. Furthermore, the duration for FFT and each video frame is 0.5 s and each trail consists of seven frames. Consequently, the temporal resolution for each trail is 7 and the visualization method can only inform us which of these video frames are more important for classification. However, this time information is not sufficient to inform cognitive theory, especially since standard EEG methods and inferences made on these methods strongly depend on the time information in the EEG signal^32^. Importantly, also the source localization method used to examine the functional neuroanatomical sources of activity critically depends on the time information^66^.

Other studies^44,51^ designed DNN architectures that work well for the multi-channel EEG signals. Their architectures are inspired by Filter bank common spatial patterns (FBCSP) algorithm^67^. In this method, at first, some temporal filters are employed and for each temporal filter, a spatial filter is employed. The spatial filters are calculated via singular value decomposition (SVD) in a way that variance in one class is maximal while in other classes is minimal. Although in FBSCP temporal and spatial filters are designed based on prior knowledge and SVD, respectively, these two kinds of filters are learned during training. After these temporal and spatial filters, some convolutional and max-pooling layers are used^44,51^. However, in EEGNet^51^ they used separable convolution, which has less parameter to estimate than ordinary convolution, which is used in other work^44^. Consequently, the model is less prone to overfit. Importantly, Schirrmeister et al.^44^ used a cropped training method to increase the accuracy of the model. This method increases the number of training data by breaking each trial into several pieces of segments (i.e., smaller than the original trail). Thus, it enlarges the training dataset, which is very useful for classification accuracy. However, this strategy is not useful in our research. Although the duration of each cropped data is the same, the time information within each of the cropped data examples cannot precisely be addressed. As a result, visualization of the model in the time domain is not possible (reliable) making it impossible to use source localization techniques. Since the EEGNet does not change the data structure we used this method for our study. Moreover, EEGNet performance has already been investigated using various event-related potential datasets^51,68^, which is not the case for other methods^43^.

### Support vector machine approach

To examine whether more simple machine learning approaches, such as support vector machines, show similar performance than the EEGNet, we used a support vector machine (SVM). For the SVM we performed the LOOS method for cross-validation. We used an SVM model with a RBF kernel and hyper-parameters are selected in the validation set through grid search among $C = \left\{ {0.01,\;0.1,\;1,\;10,\;100} \right\}\gamma = \{ 0.1,\;0,2\;,\; \ldots ,\;1,\;2,\; \ldots .,\;10\}$^43^. However, please note that our goal in this research is not to design a deep learning or machine model that can reach to best accuracy for our dataset. Instead, we want to have a model that has a good performance and for which the result of this model is interpretable in term of cognitive theory. There may exist other models that have better performance and are superior to EEGNet. However, as mentioned before, the result of these models can be very hard to interpret or they need prior knowledge about the data for feature extraction which can eliminate temporal information in EEG data.

### Source localization (sLORETA)

In this study, source localization was used to examine the source of electrical activity, which the deep learning model turned out to be most predictive for performance in one particular class of trials. For that, the standard low resolution brain electromagnetic tomography (sLORETA) algorithm was used^66^. It requires standard electrode coordinates according to the 10/10 or 10/20 system as input. The method uses a three-shell spherical head model and the covariance matrix was calculated using the single-subject’s baseline. Within this head model, the intra-cerebral volume is partitioned into 6239 voxels using a spatial resolution of 5 mm and the standardized current density is calculated for every voxel, using an MNI152 head model template. The algorithm provides a single linear solution for the inverse problem without localization bias^66,69,70^. The validity of sLORETA results have been shown in combined fMRI/EEG and TMS/EEG studies^70,71^. For the sLORETA contrasts, we performed a comparison against zero. To calculate the statistics on the sLORETA sources (contrasts), we utilized voxel-wise randomization tests with 2000 permutations and statistical nonparametric mapping procedures (SnPM). Locations of voxels that were significantly different (*p* < .05) are shown in the MNI-brain www.unizh.ch/keyinst/NewLORETA/sLORETA/sLORETA.htm. The logic of a randomization test using SnPM that if there is no condition effect, and that the labeling of the conditions is arbitrary. Using SnPM, the significance of a source is assessed by comparison with a distribution of values obtained when condition labels are permuted (i.e., 2000 times for the current data). This means that for the source reconstruction and between-condition comparisons the different source reconstruction were tested and the reported results reflect a consistent source. Activations shown in the brain represent critical t-values corrected for multiple comparisons.

### Statistics and reproducibility

The main method used was a deep learning approach. The behavioral data were analyzed using parametric tests (*t*-tests, analyses of variance, ANOVAS) using SPSS 25. *N* = 186 healthy adult volunteers participated in the study. Single-trial EEG data was used. Thus, the number of data points are: number of subjects × electrode number × sampling rate × length of the EEG intervals analysed × number of EEG epochs analyzed. For the current study, this means that ~3,348,000,000 data points were available for the deep learning procedure using the EEGNet architecture. For evaluating the classification performance of the deep learning approach, we use the “leave one out subject” (LOOS)-approach^52^ as described in the section on the deep learning procedure.

### Reporting summary

Further information on research design is available in the Nature Research Reporting Summary linked to this article.

## Supplementary information

Descriptions of additional supplementary files

supplementary data 1

Reporting Summary

## Data Availability

The datasets generated during and/or analysed during the current study are available from the corresponding author on reasonable request. Source data underlying Fig. 1, Fig. 3 and Fig. 4C can be found in Supplementary data 1.

## Code availability

We used standard software packages as described in the methods section. The EEGNet architecture can be downloaded from https://github.com/vlawhern/arl-eegmodels.
