# Supplementary material for: Applying deep learning to single-trial EEG data provides evidence for complementary theories on action control
Source: Commun Biol. 2020 Mar 9;3:112. doi: 10.1038/s42003-020-0846-z (PMC7062698; doi:10.1038/s42003-020-0846-z)
Supplement: Supplementary file 1 — Descriptions of additional supplementary files [file 42003_2020_846_MOESM1_ESM.pdf]

Descriptions of additional supplementary files

The supplemtary file (Excel tables shows the source data underlying Figure 1, Figure 3 and Figure 4C.
