# Supplementary material for: Applying deep learning to single-trial EEG data provides evidence for complementary theories on action control
Source: Commun Biol. 2020 Mar 9;3:112. doi: 10.1038/s42003-020-0846-z (PMC7062698; doi:10.1038/s42003-020-0846-z)
Supplement: Supplementary file 3 — Reporting Summary [file 42003_2020_846_MOESM3_ESM.pdf]

nature research

Corresponding author(s): Christian Beste

Last updated by author(s): 07.02.2020

Reporting Summary

Nature Research wishes to improve the reproducibility of the work that we publish. This form provides structure for consistency and transparency in reporting. For further information on Nature Research policies, see Authors & Referees and the Editorial Policy Checklist.

Statistics

For all statistical analyses, confirm that the following items are present in the figure legend, table legend, main text, or Methods section.

n/a	Confirmed	
☐	☒	The exact sample size (*n*) for each experimental group/condition, given as a discrete number and unit of measurement
☐	☒	A statement on whether measurements were taken from distinct samples or whether the same sample was measured repeatedly
☐	☒	The statistical test(s) used AND whether they are one- or two-sided<br>*Only common tests should be described solely by name; describe more complex techniques in the Methods section.*
☒	☐	A description of all covariates tested
☒	☐	A description of any assumptions or corrections, such as tests of normality and adjustment for multiple comparisons
☐	☒	A full description of the statistical parameters including central tendency (e.g. means) or other basic estimates (e.g. regression coefficient) AND variation (e.g. standard deviation) or associated estimates of uncertainty (e.g. confidence intervals)
☐	☒	For null hypothesis testing, the test statistic (e.g. *F*, *t*, *r*) with confidence intervals, effect sizes, degrees of freedom and *P* value noted<br>*Give P values as exact values whenever suitable.*
☒	☐	For Bayesian analysis, information on the choice of priors and Markov chain Monte Carlo settings
☐	☒	For hierarchical and complex designs, identification of the appropriate level for tests and full reporting of outcomes
☐	☒	Estimates of effect sizes (e.g. Cohen's *d*, Pearson's *r*), indicating how they were calculated

Our web collection on statistics for biologists contains articles on many of the points above.

Software and code

Policy information about availability of computer code

Data collection	"Presentation" (NeuroBehavioral Systems, Inc.)<br>Brain Vision Recorder Software (BrainProducts, Inc.)
Data analysis	MATLAB (MathWorks, Inc.)<br>Brain Vivision Analyzer 2 (BrainProducts, Inc.)<br>EEGNet (https://github.com/vlawhern/arl-eegmodels)<br>sLORETA software package (www.unizh.ch/keyinst/NewLORETA/sLORETA/sLORETA.htm)

For manuscripts utilizing custom algorithms or software that are central to the research but not yet described in published literature, software must be made available to editors/reviewers. We strongly encourage code deposition in a community repository (e.g. GitHub). See the Nature Research guidelines for submitting code & software for further information.

Data

Policy information about availability of data

All manuscripts must include a data availability statement. This statement should provide the following information, where applicable:

- Accession codes, unique identifiers, or web links for publicly available datasets
- A list of figures that have associated raw data
- A description of any restrictions on data availability

The data that support the findings of this study are available from the corresponding author upon reasonable request.

Field-specific reporting

Please select the one below that is the best fit for your research. If you are not sure, read the appropriate sections before making your selection.

☐ Life sciences ☒ Behavioural & social sciences ☐ Ecological, evolutionary & environmental sciences

For a reference copy of the document with all sections, see nature.com/documents/nr-reporting-summary-flat.pdf

Behavioural & social sciences study design

All studies must disclose on these points even when the disclosure is negative.

Study description	quantitative experimental
Research sample	N = 186 healthy adult volunteers between 18 and 34 years of age (mean 23.7, SD = 3.0) participated in the study. N = 106 of them were females. Participants were recruited from the TU Dresden and the Ruhr-Univeristät Bochum.
Sampling strategy	In the study, an EEG-deep learning approach was used on which depends on the available EEG data points in the entire sample. Single-trial EEG data was used. Thus, the number of data points are: number of subjects x electrode number x sampling rate x length of the EEG intervals analysed x number of EEG epochs analyzed. For the current study, this means that ~3,348,000,000 data points were available for the deep learning procedure using the EEGNet architecture (Lawhern et al., 2018).
Data collection	The software "Presentation" (version 14.9. by Neurobehavioral Systems, Inc.) was used for stimulus presentation, response recording, and sending the EEG triggers. The EEG was continuously recorded from 60 Ag/AgCl electrodes mounted in an elastic cap (EasyCap Inc.) while subjects performed the task using a BrainAmp amplifier (Brain Products Inc.) and the Brain Products recording software.
Timing	07/2011 till 11/2012
Data exclusions	No data was excluded from the analyses.
Non-participation	No participants dropped out or declined for personal reasons.
Randomization	It was a complete within subject design and there was no allocation of subjects to experimental groups.

Reporting for specific materials, systems and methods

We require information from authors about some types of materials, experimental systems and methods used in many studies. Here, indicate whether each material, system or method listed is relevant to your study. If you are not sure if a list item applies to your research, read the appropriate section before selecting a response.

Materials & experimental systems

n/a	Involved in the study	
☒	☐	Antibodies
☒	☐	Eukaryotic cell lines
☒	☐	Palaeontology
☒	☐	Animals and other organisms
☐	☒	Human research participants
☒	☐	Clinical data

Methods

n/a	Involved in the study	
☒	☐	ChIP-seq
☒	☐	Flow cytometry
☒	☐	MRI-based neuroimaging

Human research participants

Policy information about studies involving human research participants

Population characteristics	N = 186 healthy adult volunteers between 18 and 34 years of age (mean 23.7, SD = 3.0) participated in the study. N = 106 of them were females.
Recruitment	Participants were recruited from the TU Dresden and Ruhr-Universität Bochum using voluntary panel board announcements.
Ethics oversight	Ethics Commission of the Medical Faculty of the TU Dresden and the Ruhr-Universität Bochum

Note that full information on the approval of the study protocol must also be provided in the manuscript.
